# Experimental study on shear strength of saturated remolded loess

**DOI:** 10.1371/journal.pone.0271266

**Published:** 2022-07-14

**Authors:** Jie Lai, Yun Liu, Yuzhou Xiang, Wei Wang, Jiangbo Xu, Baohua Cao, Danni Zhao, Wei Wei, Han Bao, Changgen Yan, Hengxing Lan

**Affiliations:** 1 Xi’An Research Institute of High-Tech, Xi’an, China; 2 Chongqing Chengtou Road and Bridge Administration Co., Ltd, Chongqing, China; 3 Chang’an Unversity School of Highway, Xi’an, China; 4 Institute of Geographic Sciences and Natural Resources Research, Beijing, China; University of Sulaimani, IRAQ

## Abstract

Loess has the characteristics of large porosity, loose structure, uniform composition and strong collapsibility. When encountering heavy rainfall and irrigation prone to saturation, resulting in loess landslides, roadbed subsidence and dam instability. In order to study the effect of dry density and shear rate on the shear strength of saturated remolded loess, the consolidated undrained (CU) test was carried out in Yan’an City by using SLB-6A stress-strain controlled triaxial shear permeability test instrument. The shear rate, confining pressure and dry density were controlled during the test. The dry densities of the samples were 1.5 g / cm3, 1.6 g / cm3 and 1.7 g / cm3, respectively. CU tests of saturated remolded loess were carried out at different shear rates under the confining pressures of 100 kPa, 150 kPa and 200 kPa, respectively. It is found that the stress-strain curve of saturated remolded loess gradually moves upward with the increase of dry density. With the increase of dry density, the cohesion and internal friction angle of remolded saturated loess samples increase. At the same shear rate, with the increase of dry density, the deviatoric stress of the specimen increases significantly.

## 1 Introduction

**Fig 1:**
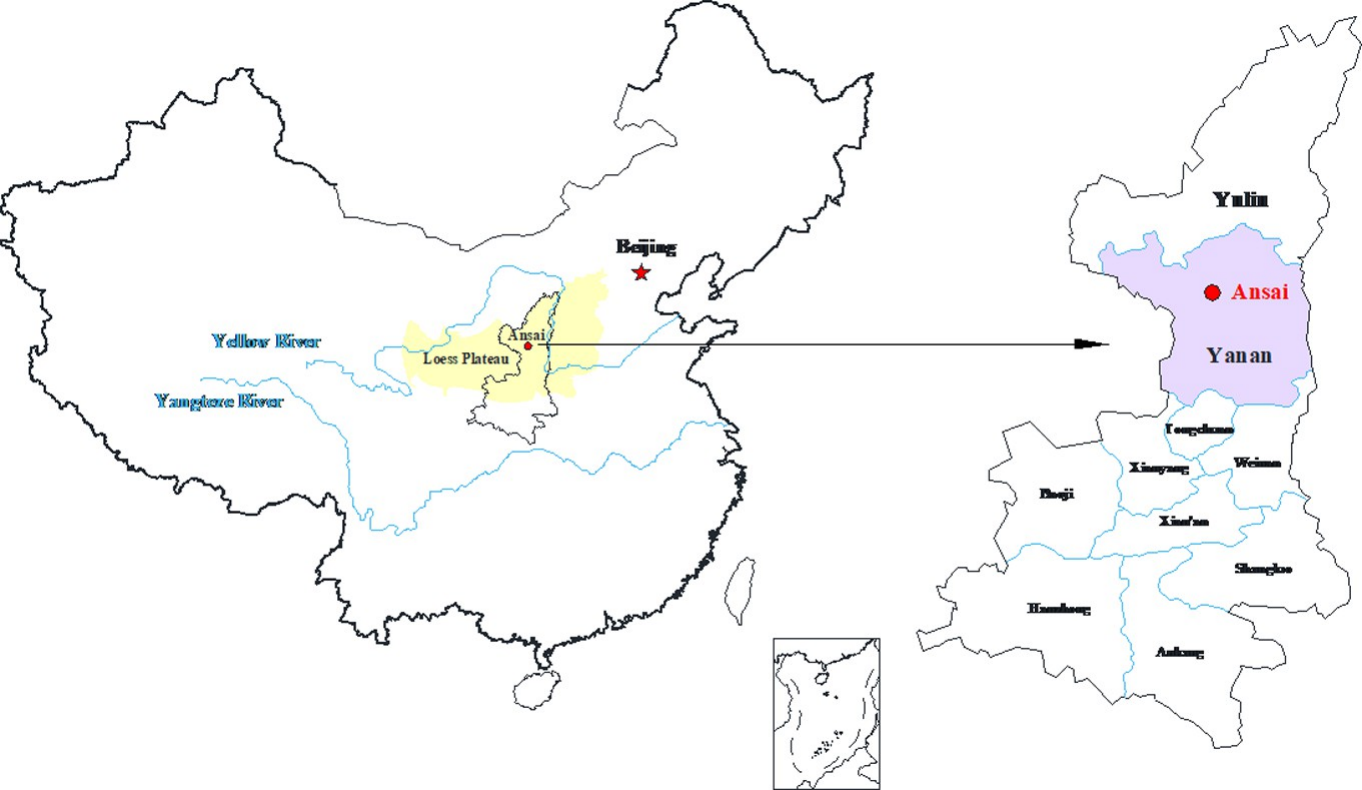
Distribution of loess in China.

Loess is widely distributed in northwestern China, forming a loess area in Gansu, Shaanxi, Shanxi and other provinces as shown in Fig 1. In recent years, with the development of the national economy in the western loess region, large-scale leveling of the mountain to build land and subgrade engineering project have increased dramatically. Due to its unique engineering properties, loess has caused landslides, dam instability [1], and roadbed collapsibility. The most serious geological disasters in the northwest region pose a serious threat to the safety of people’s lives and property [1]. Water infiltration and fine-grained soil backfilling lead to cracks on the road shoulder [2]. For example, large-scale agricultural irrigation has led to the frequent occurrence of loess landslides [3]. The influence of rainfall intensity on landslide was analyzed to build a rainfall threshold model [4]. The improved frequency ratio method is used to evaluate the sensitivity of landslides [5]. Sensitivity analysis is conducted on the landslide stability of Xiaojiang watershed in Yunnan Province [6], and a new landslide analysis model is proposed based on GIS [7]. Zizhou County of Yulin City is a typical loess plateau. According to the survey, there are 128 geological disasters in the county, including 78 landslides and 44 collapses [8]. Therefore, based on laboratory tests, the influence of various factors on the shear strength of saturated remolded loess is discussed, which is of great significance for revealing the mechanism of geological hazard induced by loess saturation.

At present, many scholars have carried out corresponding research on the influencing factors of the shear strength of loess and have made some achievements. The shear resistance of the contact between particles and the bite of the particles are important reasons for the shear resistance of the soil in the deformation [9]. The effect of bulk density and matrix potential on the shear strength of soil was studied (Cruse et al. [10] investigated the effect of bulk density and matrix potential on soil shear strength). Soil shear strength is defined as the resistance to a shear failure caused by continuous shearing of soil particles or soil masses [11]. The combination of cohesive binding factors of clay and organic matter in soil subjected to varying wetting conditions exert a strong influence on the resistance of the soil to sustained shearing stresses [12]. The influencing factors of soil shear strength after rainfall was studied. (Watson et al. [13]explored the role of rainfall on soil shear strength). The relationship between the soil-water characteristic curve and the shear strength of unsaturated soil is described, which is related to matrix suction [14]. An empirical, analytical model was developed to predict the shear strength in terms of soil suction. In the field of unsaturated soil research, The expression of effective stress of unsaturated soil similar to the principle of effective stress of Terzaghi, and extend the criterion of shear strength of saturated soil to unsaturated soil [15]. The shear strength formula is raised for unsaturated soils. Considering the influence of suction on the strength of unsaturated soils, and two different parameters were selected. The difficulty of the shear strength formula proposed by the above two scholars is that the effective stress parameter *χ* and the suction internal friction parameter *φ*_*b*_ are uncertain, which limits the practical application [16]. Based on the consideration of the interaction between micro-particles and the effective stress of Terzaghi, the theory of soil stress was proposed, which clarified the physical meaning of soil cohesion from the microscopic point of view, that is, the frictional strength generated by the suction stress in the soil during the shearing process [17]. Influence of the history of shear paths on yield and failure surfaces is discussed, and the position of yield was found to be independent of the previous shearing path history of the soil and occurred at points that corresponded to a yield surface defined for the current shearing path direction [18]. A hyperbolic function model based on the experimental data was form, which enriched the theoretical system of nonlinear shear strength function [19]. The steady strength of saturated loess was studied under the stress-controlled undrained consolidation triaxial = tests. There are two typical stress-strain behaviors of saturated loess: steady-state behavior and quasi-steady state. In the majority of situations, it exhibits steady- state behavior. Only loose loess exhibits quasi-steady -state behavior [20]. The water migration in loess samples is researched based on the damage of water in subgrade [21]. Soil samples of loess slope arecollected in Longdong, and shear test was carried out. It was found that the anisotropy of loess had little effect on friction angle, and had a certain effect on cohesion [22]. An experimental study was presented to investigate the influence of sand and water content on the small-strain shear modulus (G_0_) of Yan’an loess, and found that the roundness of soil particles can reduce the shear wave velocity [23]. Strain softening exists in fault gouge as well as loess through the cyclic shear test [24]. The effects of dry density on the strength and deformation characteristics of Q_2_ remolded loess is analyzed. The test results showed that the deviatoric stress-axial strain curves of the specimens exhibited strain hardening under different dry densities. As the dry density increases, the deviatoric stress of the sample increases remarkably, and the hardening tendency increases gradually [25]. Quantitative parameters are raised to reflect the structure of remolded loess and undisturbed saturated loess based on the triaxial test [26]. *UU(unconsolidated undrained)* and *CU* (Undrained consolidation) tests on saturated undisturbed loess are carried out, and obtained the relationship between the initial tangential modulus, shear strength and stress path of the undisturbed loess under different confining pressures [27]. Leng [28] considered the influence of confining pressure on the shear strength of saturated loess and gave the corresponding strength reduction formula. Saturated loess with different dry density through different stress paths are researched, and found that under the same path, the stress-strain, the size of dry density does not affect the shape of its curve, but the strength and anti-destructive ability of the soil [29]. The modified TFB-1 unsaturated soil stress-strain control triaxial apparatus is used to carry out saturation test and *CU* test on the remolded soil samples of Malan loess in the Dangchuan area, and the study found that the shear strength of saturation remolded loess under the same confining pressure increases first and then decreases with the increase of shear rate. Under the same shear rate, the shear strength of saturated remolded loess increases with the increase of confining pressure. CTC and RTC tests are conducted on saturated remolded loess and saturated undisturbed loess, and it is found that the shear strength of saturated undisturbed loess under low confining pressure is higher than that of saturated remolded loess, and the opposite under high confining pressure [30]. Reinforcement effect of sand dunes by grouting soil with silica fume slurry. The study shows that the erosion amount of 67% water stabilizer slurry will be reduced by about 70% according to the total mixed weight, and the stability effect will be enhanced with the increase of L-SF slurry [31]. The leaching characteristics of three different gypsum soil samples were studied. The change of microstructure was studied by combining X-ray fluorescence and scanning electron microscopy (SEM) analysis. It was found that the solubility of gypsum minerals was proportional to gypsum content and leaching cycle [32]. Model tests were carried out on disturbed natural gypsum soil with 18%, 30% and 55% gypsum ratios. The variation of suction, settlement and total vertical stress with time and the effect of wetting on volume change of unsaturated gypsum soil were studied [33].

In conclusion, researchers both at the local and international have conducted research on remolding saturated loess and have obtained corresponding results in shear rate and confining pressure on the shear strength of loess. Moreover, no appropriate theoretical system has been developed. It is difficult to obtain the undisturbed loess in reality, so laboratory tests are carried out on remolded loess in the Yan’an area. First, water head saturation and back pressure saturation are used to saturate the sample, and then the CU test is carried out directly on the sample. It is of practical significance and theoretical value to study the influence of various factors on the shear strength of remolded loess by combining the method of water head saturation, back pressure saturation and CU tests.

## 2 Soil sample and testing methodology

### 2.1 Materials

**Fig 2:**
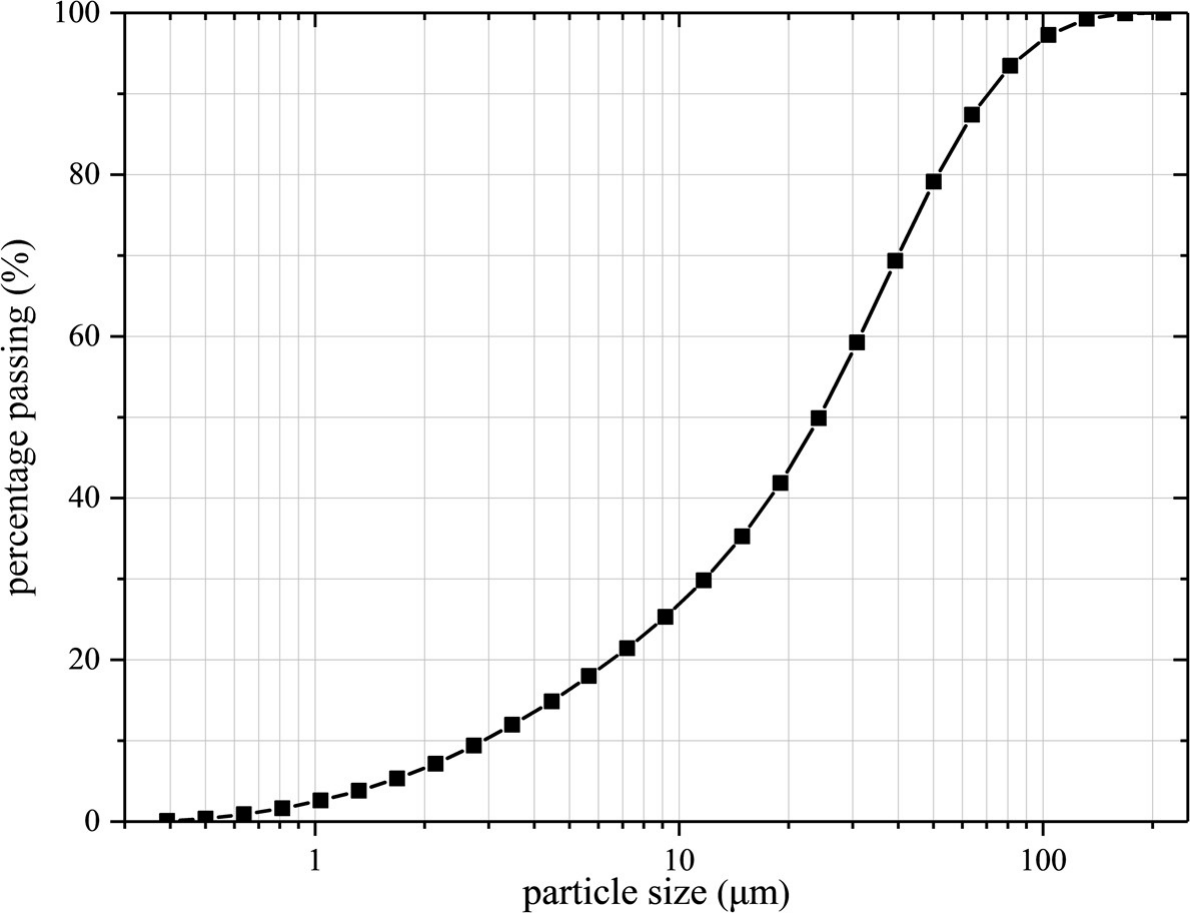
Granular gradation curve of loess.

The test soil was a brownish -yellow silty clay and taken from the Ansai area of Yan’an, Shaanxi Province. Before the preparation of the test soil sample, the loess is first naturally dried and crushed, then screened by a 2 *mm* sieve and stored in a glass tank for reserve. Its physical properties are shown in Table 1. The granular gradation curve of the soil is shown in Fig 2. The size of the remolded soil sample in this test was 39.1*mm* × 80*mm*, wherein the sample diameter was 39.1 *mm*, and the sample height was 80 *mm*.

**Table 1:** Basic physical parameters of soil samples.

Moisture content/%	Dry density/(*g / cm*^*3*)^	Percentage of soil particles at different particle sizes/%			
		< 0.075	0.075–0.25	0.25–0.5	0.5–2
14.97	1.557	88.73	12.27	0	0

### 2.2 Experimental method

The device used in this test is an SLB-6A stress-strain controlled triaxial shear permeability tester. Its confining pressure range is 0–1 *MPa*, and the strain of clay is 0.05%-0.1% per minute. It can be controlled and date are collected by the computer. The test process is divided into three stages: water head saturation stage, back pressure saturation stage and *CU* stage. Firstly, 10 *kPa* confining pressure is set to saturate the loess sample, and the air in the rubber film is discharged, the first confining pressure and back pressure are applied after the water head saturation is over to conduct the back pressure saturation, and each stage is increased by 10 *kPa*. In the whole saturation test process, the water head saturation time is 3–4 hours and the back pressure saturation time is 5–6 hours, which greatly shortens the saturation time and the saturation effect is obvious. At the end of the saturation test, the B value is tested, that is, the back pressure remains unchanged, and the confining pressure is increased by 40*kPa*. The increment of pore water pressure caused by the increment of confining pressure *(Δσ*_*3*_*)* is *Δu*.


$$B=\Delta u/\Delta \sigma_{3}$$ (1)

Obtaining a B value of unity during the actual test process is complicated. When the B value reaches 95%, it can be considered that the sample is saturated. the back pressure valve is closed after the saturation test meets the test requirements. (The reasons are as follows. According to the results of triaxial compression tests, the relationship between the pore pressure and the large and small principal stresses under axisymmetric stress can be established by referring to the pore pressure coefficients A and B. By the effective stress formula and the elastic theory, When the elastic modulus and Poisson ’ s ratio of elastic material are E and μ, the change of soil volume formula can be obtained when the stress and shear stress are equal.

$$Β=\frac{1}{1+n\frac{Cv}{Cs}}$$ (2)

where n represents the porosity of soil, C_v_ represent three to pore volume compressibility, C_s_ on behalf of the three to the volume compression coefficient of soil skeleton. Reason for saturated soil, completely filled with water in the pores, due to the compressibility of water is much lower than the compressibility of soil skeleton, $\frac{\text{Cv}}{\text{Cs}}$→0, and B = 1; For dry soil, $\frac{\text{Cv}}{\text{Cs}}$ →∞, therefore, B = 0; For unsaturated soil, 0<B<1, the less saturated the soil, the smaller the B value.) Three dry densities of soil samples were used in experimental works 1.5 g/cm3, 1.6 g/cm, and 1.7 g/cm3. The CU test was carried out under the confining pressure of 100*kPa*, 150 *kPa* and 200 *kPa*, and the shear rate of 0.04 *mm/min*, 0.08 *mm/min*, 0.16 *mm/min* and 0.4 *mm/min* respectively. The relationships between dry density and cohesion, dry density and internal friction angle, shear rate and cohesion, shear rate and internal friction angle, and shear rate and internal friction angle can be obtained by controlling the shear rate and confining pressure, respectively. Table 2 below depicts the test plan.

**Table 2:** Test design scheme.

Dry density/(*g/cm*^*3*^)	Confining pressure/*kPa*	Shear rate/*mm•min*^*-1*^
1.5	100、150、200	0.04、0.08、0.16、0.4
1.6	100、150、200	0.04、0.08、0.16、0.4
1.7	100、150、200	0.04、0.08、0.16、0.4

## 3 Analysis of test results

Pore water pressure and axial strain are expressed by *u* and *ε* respectively, where *p* is the center coordinate of the circle of molar stress, *q* is the radius of the mohr’ s stress circle, and *σ*_*1*_ and *σ*_*3*_ are the major and minor principal stresses respectively. The values of *p* and *q* are shown in Table 3.


$$p=\left(\sigma_{1}+\sigma_{3}\right)/2$$ (3)

$$p'=\left(\sigma_{1}+\sigma_{3}\right)*0.5-u$$ (4)

$$q'=q=\left(\sigma_{1}-\sigma_{3}\right)/2$$ (5)

**Table 3:** *p&p’*and *q* values at different dry densities and shear rates.

Dry density/*(g•cm*^*-3*^*)*	Shear rate v/*mm•min*^*-1*^	Confining pressure/*kPa*	*p*	*q&q’*	*p’*	*σ*_*1*_	*u*
1.5	0.04	100	146.20	46.20	67.20	192.4	79
		150	222.80	72.80	102.80	295.6	120
		200	284.80	84.80	115.80	369.60	169
	0.08	100	167.65	67.65	95.65	235.30	72
		150	231.80	81.80	99.80	313.60	132
		200	284.40	84.40	125.40	368.80	159
	0.16	100	146.25	46.25	67.25	192.50	79
		150	214.10	64.10	96.10	278.20	118
		200	294.20	94.20	122.20	388.40	172
	0.4	100	141.10	41.10	63.10	182.20	78
		150	207.65	57.65	82.65	265.30	125
		200	291.20	91.20	120.20	382.40	171
1.6	0.04	100	165.80	65.80	102.80	231.60	63
		150	241.55	91.55	131.55	331.10	110
		200	329.25	129.25	172.25	458.50	157
	0.08	100	217.25	117.25	145.25	334.50	72
		150	299.55	149.55	187.55	449.10	112
		200	370.00	170.00	214.00	540.00	156
	0.16	100	170.10	70.10	111.10	240.20	59
		150	226.25	76.25	122.25	302.50	104
		200	302.80	102.80	149.80	405.60	153
	0.4	100	203.95	103.95	132.95	307.900	71
		150	295.00	145.00	166.00	440	129
		200	347.95	147.95	197.95	495.90	150
1.7	0.04	100	222.45	122.45	176.45	344.90	46
		150	276.60	126.60	206.60	403.2070	70
		200	394.80	194.80	281.80	589.60	113
	0.08	100	276.75	176.75	234.75	453.50	42
		150	346.55	196.55	278.55	543.10	68
		200	417.10	217.10	330.10	688.20	114
	0.16	100	216.95	116.95	175.95	333.90	41
		150	302.85	152.85	225.85	455.70	77
		200	364.25	164.25	247.25	528.50	117
	0.4	100	260.15	160.15	220.15	420.30	40
		150	338.10	188.10	275.10	526.20	63
		200	401.05	201.05	283.05	602.10	118

### 3.1 Effect of dry density and shear rate on deviation stress

**Fig 3:**
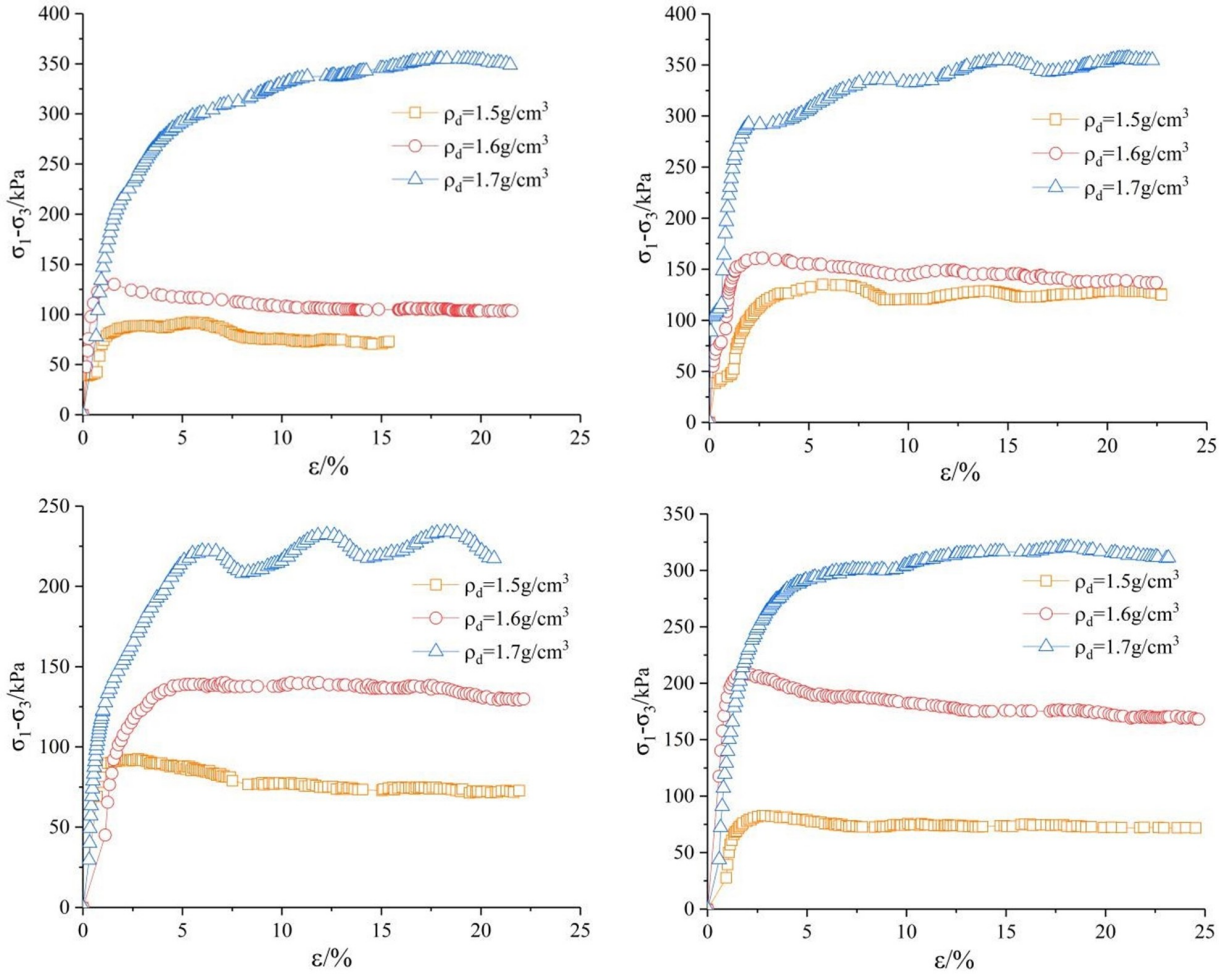
*σ*_*1*_*-σ*_*3*_*~ε* curve under confining pressure of 100*kPa*. (a) Shear rate *v* = 0.04 *mm/min*. (b) Shear rate *v* = 0.08 mm/min. (c) Shear rate *v* = 0.16 *mm/min*. (d) Shear rate v = 0.4 *mm/min*.

**Fig 4:**
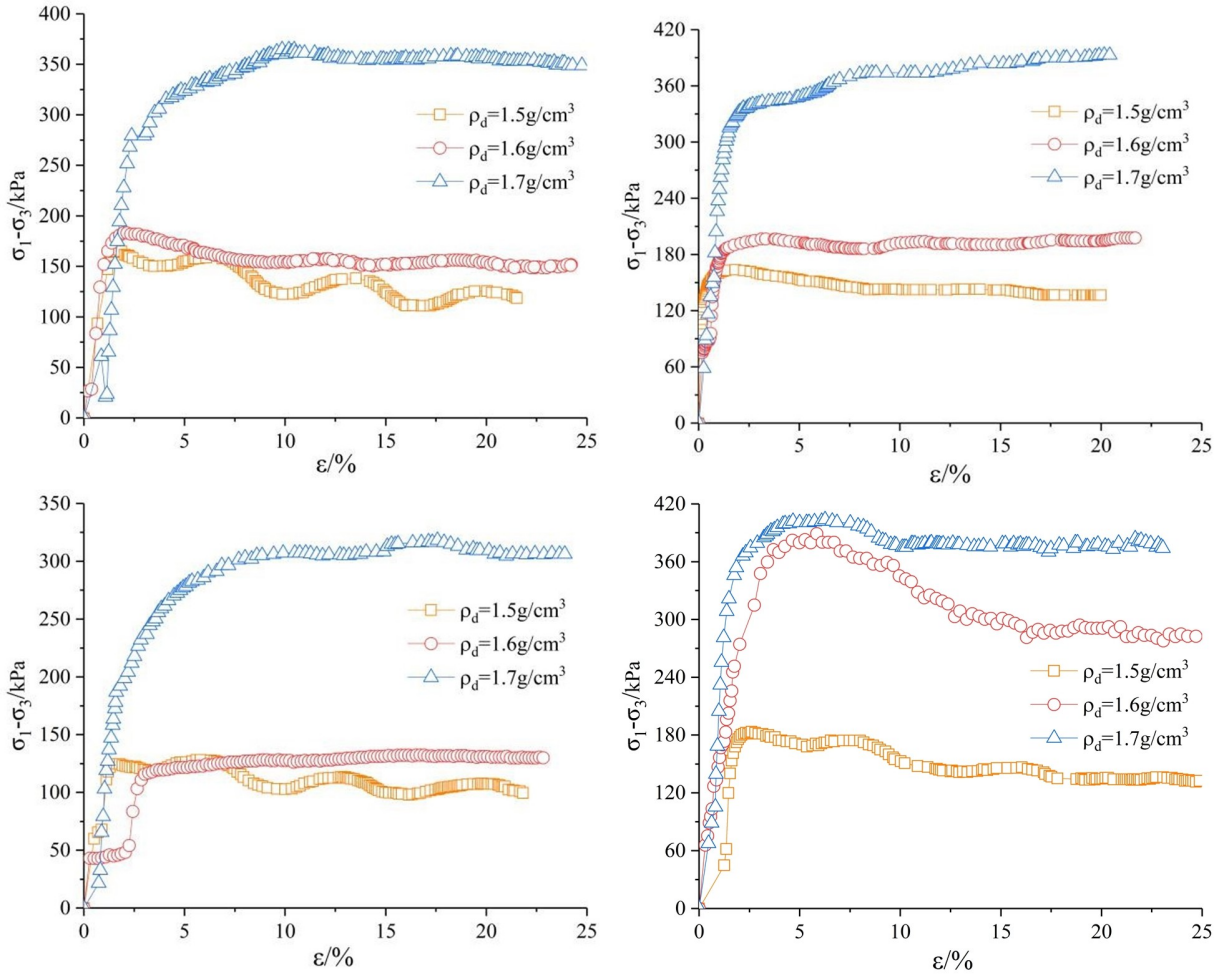
*σ*_*1*_*-σ*_*3*_*~ε* curve under confining pressure of 150*kPa*. (a) Shear rate *v* = 0.04 *mm/min*. (b) Shear rate *v* = 0.08 *mm/min*. (c) Shear rate *v* = 0.16 *mm/min*. (d) Shear rate *v* = 0.4 *mm/min*.

**Fig 5:**
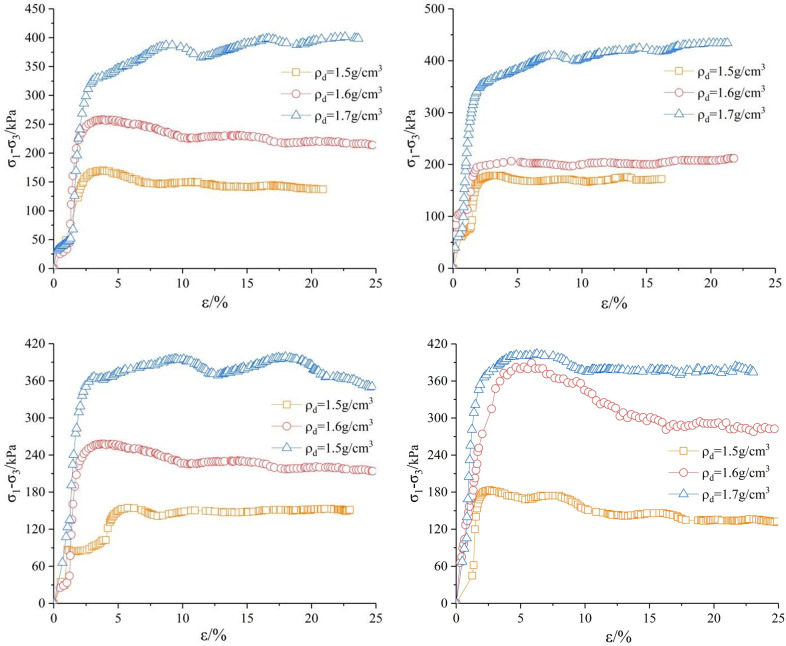
*σ*_*1*_*-σ*_*3*_*~ε* curve under confining pressure of 200*kPa*. (a) Shear rate *v* = 0.04 *mm/min*. (b) Shear rate *v* = 0.08 *mm/min*. (c) Shear rate *v* = 0.16 *mm/min*. (d) Shear rate *v* = 0.4 *mm/min*.

Figs 3–5 show the stress-strain relationship. It can be seen that the deviatoric stress of the sample increases significantly at the same shear rate with the increase of dry density. The deviatoric stress-axial strain curves of the sample when *ρ*_*d*_ = 1.7 *g/cm*^*3*^ are strain hardening. When *ρ*_*d*_ = 1.5 g/cm^3^ and *ρ*_*d*_ = 1.6 *g/cm*^*3*^, the deviatoric stress-axial strain curves of soil samples show two forms: weak softening and softening, and the softening is obvious under low confining pressure. Generally, the peak value of *σ*_*1*_*-σ*_*3*_ is taken as the failure point, but the deviatoric stress at 15% axial strain is taken as the failure point when there is no peak value. The deviatoric stress- axial strain curve of saturated remolded loess can be divided into two stages. In the first stage, the deviatoric stress rapidly reaches the peak point with the strain increase, and then enters the second stage. The deviatoric stress tends to decrease slowly with the increase of strain, and approaches a certain value when the strain reaches 20%, which is no obvious stress drop in the whole stage.

**Fig 6:**
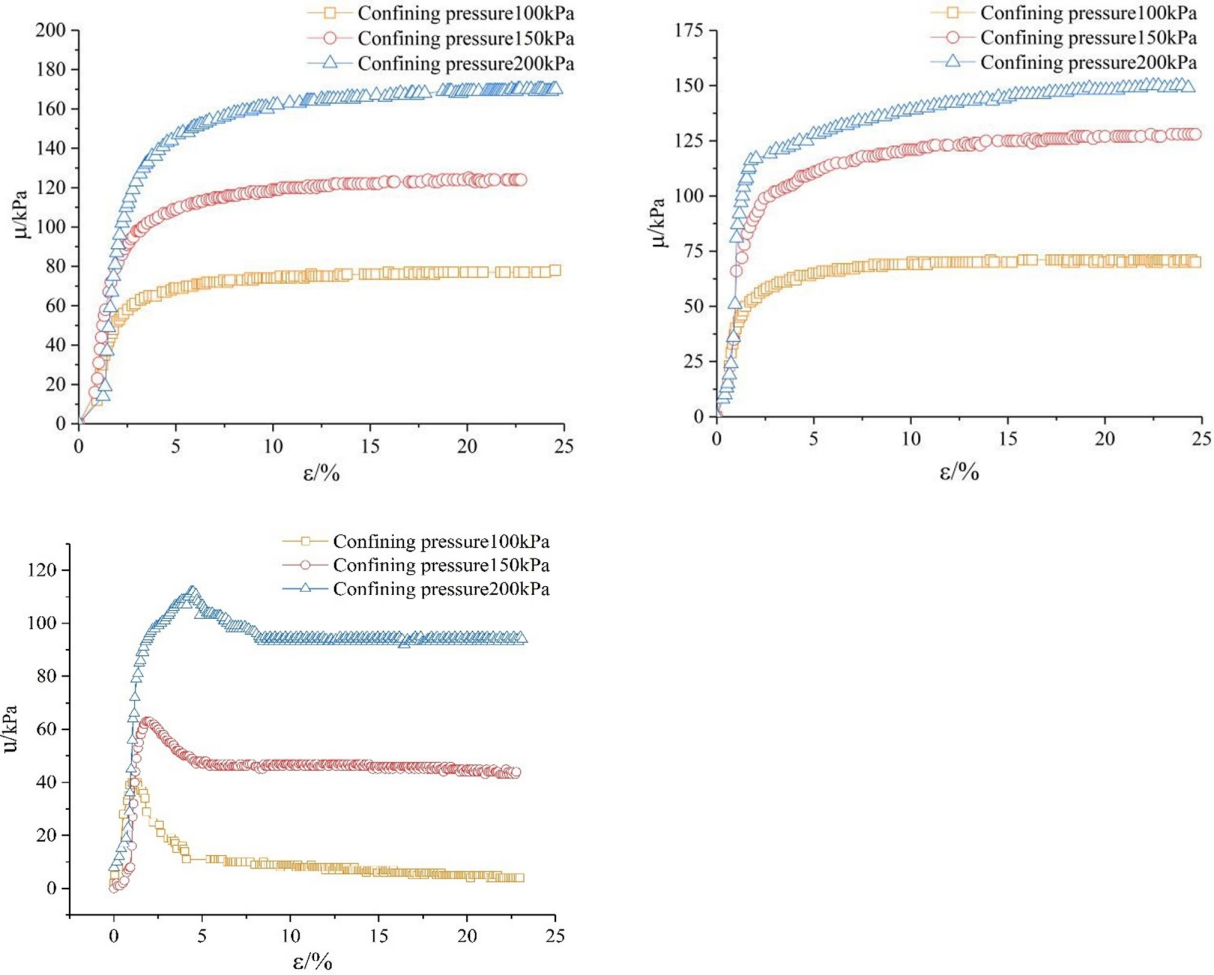
Pore water pressure *u* curve with shear rate of 0.4mm/min at dry density of 1.5g/cm^3^, 1.6g/cm^3^, 1.7g/cm^3^. (a) *ρ*_*d*_ *=* 1.5 *g/cm*^*3*^. (b) *ρ*_*d*_ *=* 1.6 *g/cm*^*3*^. (c) *ρ*_*d*_ = 1.7 *g/cm*^*3*^.

The curve of pore water pressure versus strainis shown in Fig 6. When the soil sample reaches a saturation state, the free water fills the whole soil pore. Because water can not be compressed and does not bear shear force, it exists between soil particles as a barrier zone, which affects the contact between particles. Most of its occlusive friction is offset by the lubrication of pore water, which reduces the cohesion between soil particles and increases the irrecoverable plastic strain in the sample, and finally make the strength reduce. When the soil’s dry density is higher, it has fewer pores and less free water in those pores, which reduces occlusive friction between soil particles and boosts shear strength. When the dry density value is high, the soil sample is in a dense state, and the peak value of the deviatoric stress-axial strain curve is large, while the pore water pressure tends to appear negative, showing a large dilatancy, and thus a lower pore water pressure.

**Fig 7:**
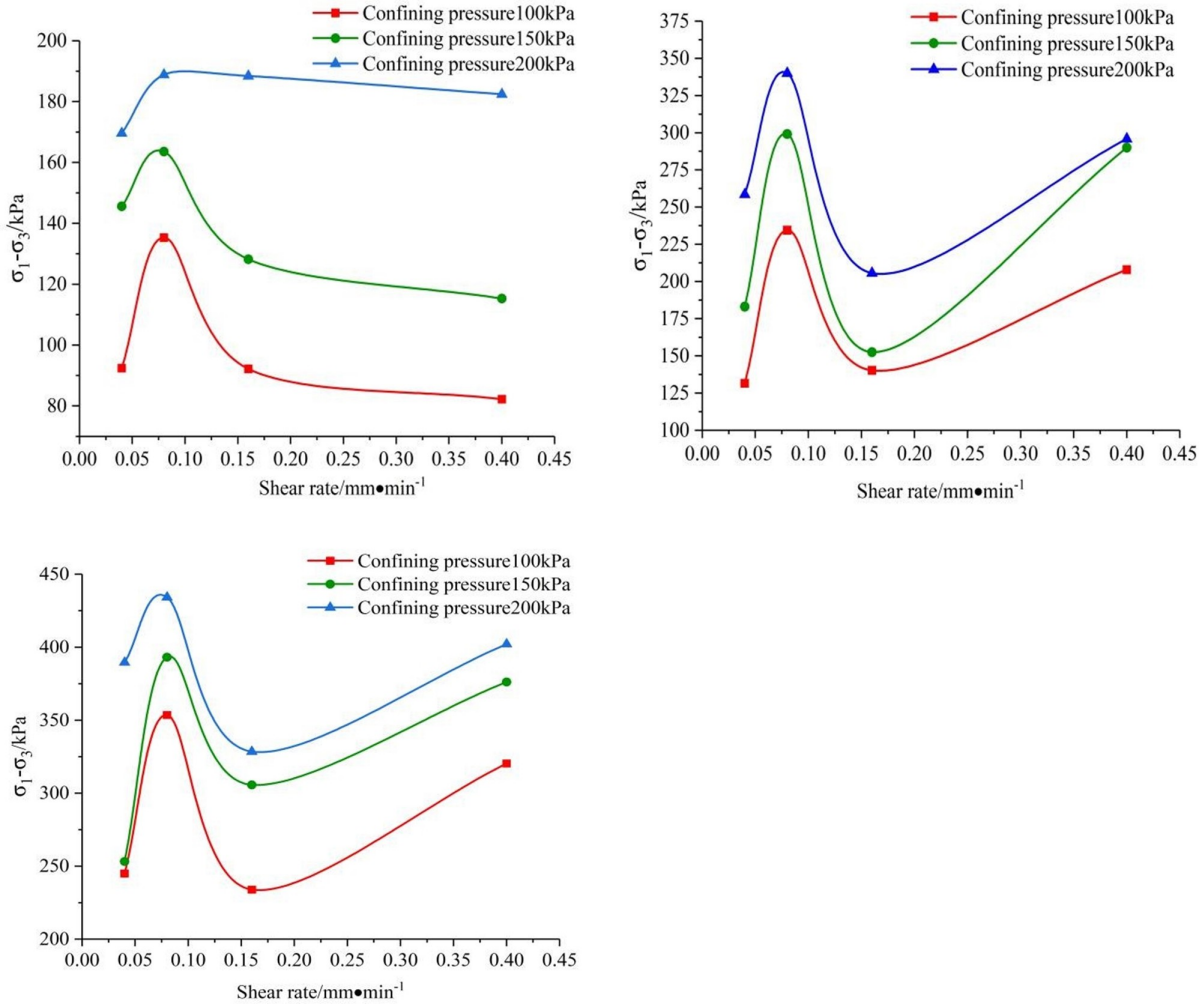
The relation between deviatoric stress and shear rate under different confining pressures. (a) *ρ*_*d*_ = 1.5 *g/cm*^*3*^. (b) *ρ*_*d*_ = 1.6 *g/cm*^*3*^. (c) *ρ*_*d*_ = 1.7 *g/cm*^*3*^.

With the shear rate as the transverse axis and The difference between large and small principal stresses as the longitudinal axis to obtain the relationship curve between the deviatoric stress and the shear rate under different confining pressures, which is drawn from Fig 7. It can be seen that the shear strength of saturated remolded loess under the same confining pressures obviously affected by the shear rate, and the relationship between the shear strength and the shear rate is not a simple monotone function. It is generally believed that the deviatoric stress increases with the increase of strain rate, which is different from the experimental results. When the dry density is *ρ*_*d*_ = 1.5 *g/cm*^*3*^, it can be shown from Fig 7A that the shear strength increases first and then decreases as the shear rate increases, and there is a significant critical shear rate. As the confining pressure increases, the overall appearance increases and then decreases. The softening behavior at a high strain rate is noticeably different from that at a low strain rate. This phenomenon may be related to the thixotropy of loess. In high stain rate, structure of loess clay may be destroyed,which may be the reason of strain rate softening. When the shear rate is low, the loess structure is not obviously damaged, and the partial strength of the loess loss can be recovered. At the higher shear rate, the loess structure is destroyed, and the strength of loss is relatively large, which leads to the appearance of low shear strength at a high shear rate (Chen et al., 2010). When the dry density is equal to *ρ*_*d*_ = 1.6 *g/cm*^*3*^ and *ρ*_*d*_ = 1.7 *g/cm*^*3*^, it can be revealed from Fig 7B and 7C When the shear rate is increased, the deviatoric stress increases first, then decreases, and then rises again. It can be inferred that when the shear rate is small, the structural properties of loess are not destroyed and some strength of loess can be restored, but the internal of loess clay tends to be destroyed when the test is about 0.16*mm/min*, and the strength of loess recovers slowly, so the curve has a downward trend. The permeability coefficient of loess decreases relatively when the dry density is relatively large, and uneven pore pressure will occur at an excessively high shear rate, which limits the selection range of shear rate, thus the thixotropy of loess can not be expressed. The effect of shear rate of loess is controlled by many factors, such as dry density and confining pressure. The strain -softening phenomenon is weaker in soils with relatively low thixotropy, and the shear strength increases with the increase of strain rate. When the dry density is large, the strength of saturated loess increases relatively with time in the shear process. As a result, with the increase of shear rate, the thixotropy of loess did not make the structural properties of loess be damaged obviously, and some strength of loess could be restored quickly. Thus, the deviatoric stress increased with the increase of shear rate between 0.16 *mm/min* and 0.4 *mm/min*.

### 3.2 Effect of shear rate and dry density on stress path

**Fig 8:**
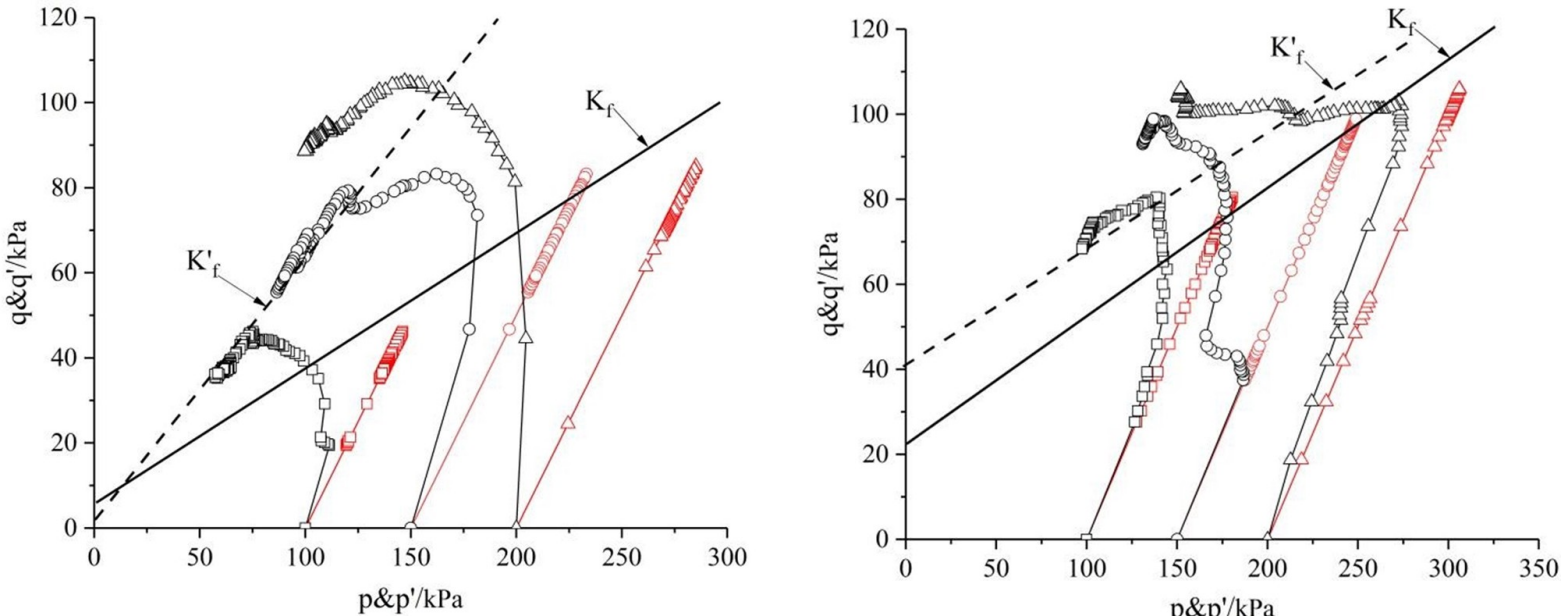
*p~q* curve with *ρ*_*d*_ = 1.5*g/cm*^*3*^ and *ρ*_*d*_ = 1.6*g/cm*^*3*^. (a) Shear rate *v* = 0.04*mm/min*. (b) Shear rate 0.04*mm/min*.

**Fig 9:**
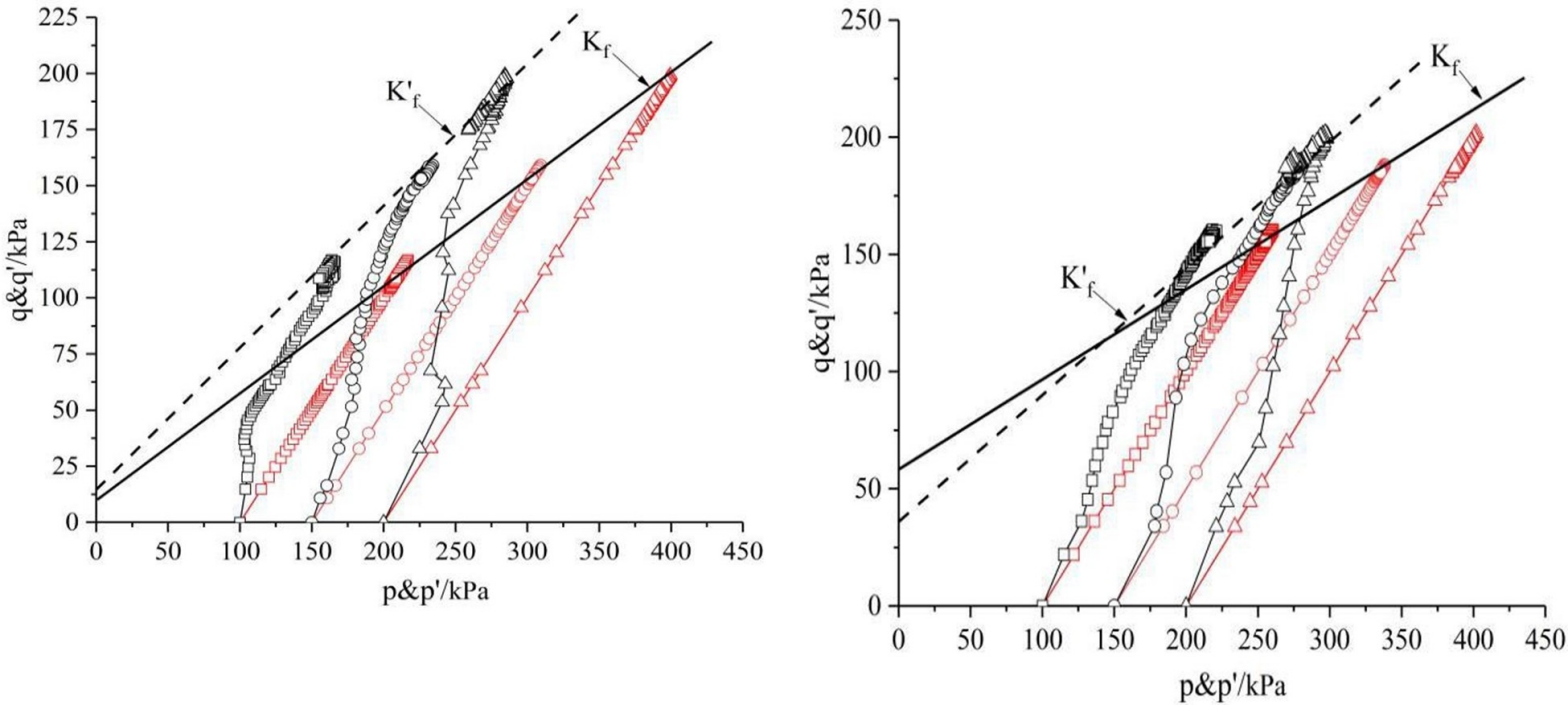
*p~q* curve with *ρ*_*d*_ = 1.7 *g/cm*^*3*^. (a) Shear rate *v* = 0.16 *mm/min*. (b) Shear rate *v* = 0.4 *mm/min*.

Figs 8A and 8B and 9A and 9B are the *p~q* and *p’~q* curves of saturated remolded loess under confining pressure of 100*kPa*, 150*kPa* and 200*kPa* respectively. The failure principal stress lines *K’*_*f*_ of effective stress path and failure principal stress lines *K*_*f*_ of total stress path are the aggregation of all the points in limit equilibrium stress state to show in *p’-q* coordinate system and *p-q* coordinate system respectively. Only the most significant representative graphs are chosen for this paper. When undrained shear occurs,with the deviatoric stress increases, the total stress path of the point in the sample develops upward in a straight line with a certain angle to the *p*-axis. With undrained shearing, excess pore water pressure *u* is generated in the specimens. The pore pressure coefficient A changes continuously during shear for saturated soil, and the effective stress path is a curve.

$$u=A\times \left(\sigma_{1}-\sigma_{3}\right)$$ (6)

p'=p−u (7)

The undrained shear stress path of saturated remolded loess under low confining pressure is obviously different from that under high confining pressure. The effect of larger dry density on the stress path of saturated remolded loess is greater than that of lower dry density. As shown in Fig 9A and 9B, the shapes of the curves are different under different shear rates. With the increase of confining pressure, the initial stage of the stress path is more and more perpendicular to the *p*-axis. With the increase of strain, the stress path first reaches its peak strength at different shear rates, and then tends to a narrow region in the stress space. This phenomenon shows that the concept of critical state is still applicable to remolded loess related to shear rate.

### 3.3 Variation of shear strength index with shear rate and dry density

The values of total residual strength parameters *c*, *φ* and effective residual strength parameters *c’*, *φ’* of the saturated remolded loess in *CU* test can be obtained by linear fitting of *p~q* and *p’~q* curves. The intercept between the fitting line and the ordinate is *c*_*q*_, and the slope is *φ*_*q*_. There is a relational expression between *c*_*q*_, *φ*_*q*_ and cohesion *c*, friction angle *φ*(8), (9), and the total residual shear strength index and effective residual shear strength index of saturated remolded loess at different shear rates are shown in Table 4.


$$sin\varphi =tan\varphi_{q};$$ (8)

$$c=c_{q}/cos\varphi ;$$ (9)

**Table 4:** Effective residual shear strength and total residual strength under different dry density and shear rate conditions.

Dry density /(*g•cm*^*-3*)^	Shear rate/*mm•min*^*-1*^	*c/kPa*	*φ /(°)*	*c’/kPa*	*φ’/(°)*
1.5	0.04	6.940	16.320	24.286	29.406
	0.08	6.199	17.999	23.451	33.025
	0.16	6.874	16.559	24.152	29.736
	0.4	8.562	15.308	24.358	28.164
1.6	0.04	12.778	20.121	28.174	31.870
	0.08	28.307	23.891	50.223	34.960
	0.16	7.395	18.844	17.148	32.480
	0.4	37.762	20.060	54.674	31.064
1.7	0.04	33.864	23.079	30.015	32.208
	0.08	40.877	27.580	42.653	35.170
	0.16	29.252	23.578	29.819	33.642
	0.4	55.076	23.453	55.101	31.668

**Fig 10:**
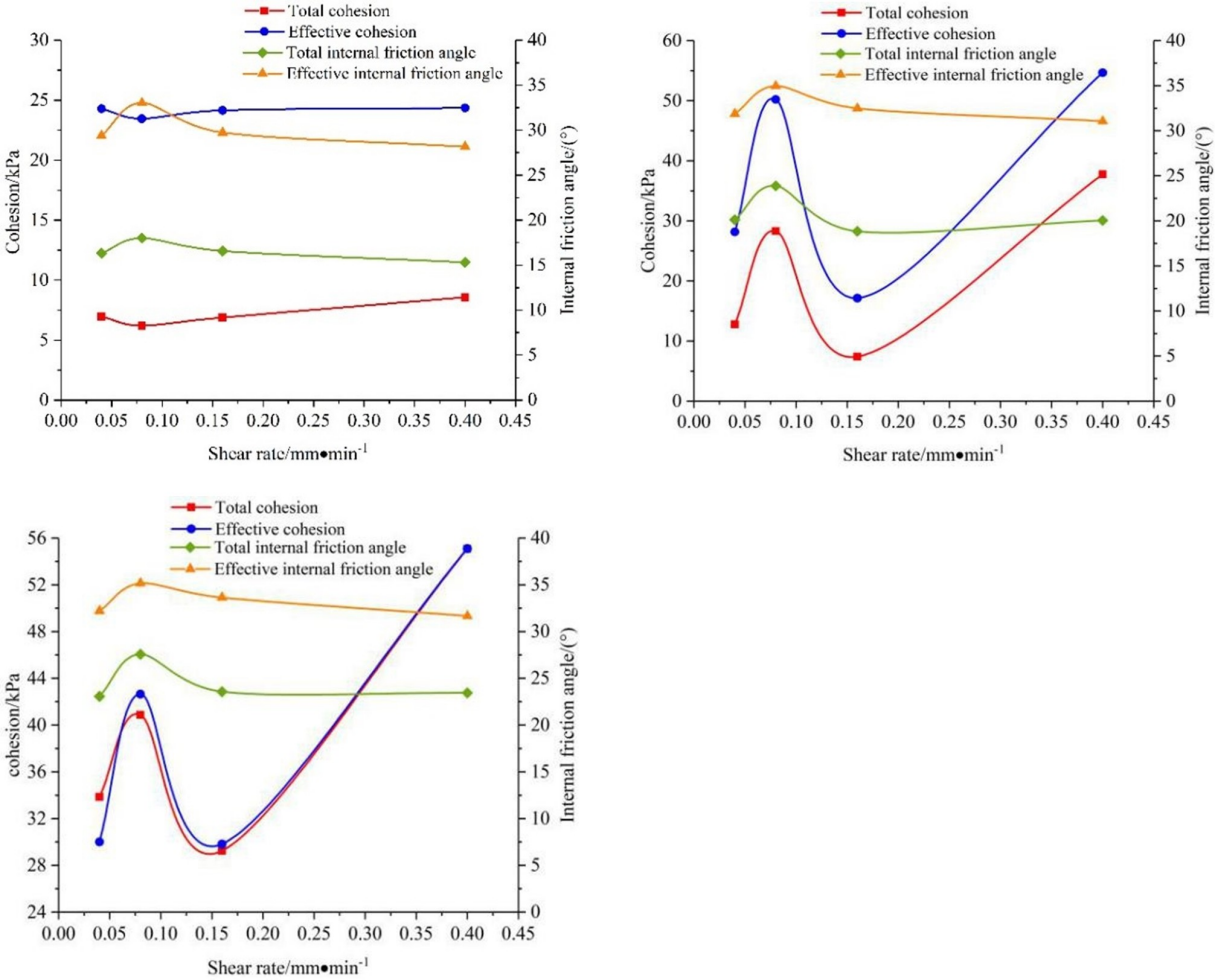
Relationship between cohesion, internal friction angle and a shear rate of saturated remolded soil samples. (a) *ρ*_*d*_ *= 1*.*5 g/cm*^*3*^. (b) *ρ*_*d*_ *= 1*.*6 g/cm*^*3*^. (c) *ρ*_*d*_ = 1.7 *g/cm*^*3*^.

The shear rate is the horizontal axis, and the shear strength index of the saturated remolded loess sample is the vertical axis. The relationship between the cohesion, the internal friction angle of the remolded soil sample and the shear rate is plotted. As shown in Fig 10A–10C, the total and effective internal friction angles of saturated remolded loess under different dry densities, always increase first and then decreases with the increase of shear rate. When dry density is equal to 1.5*g/cm*^*3*^, the cohesion decreases first and then increases with the increase of shear rate. When dry density is equal to 1.6*g/cm*^*3*^ and equal to 1.7*g/cm*^*3*^, with the increase of shear rate, the cohesion first increases at 0.08*mm/min*, after that, the cohesive force decreases first and then increases. This phenomenon may be caused by swelling of saturated soil samples during the *CU* test. The cohesion of soil is composed of original cohesion and solidified cohesion. The former comes from the electrostatic force and van der Waals force between particles, that is to say, it is determined by the gravity between particles molecules in the soil, which is related to the density of the soil. The latter depends on the cementation of cementitious materials between particles. The test soil sample is fine-grained silty loess, and the internal friction angle of the fine-grained soil is affected by density, particle gradation, particle shape, etc. The denser the soil, the smaller the roundness, the stronger the bite cooperation and the greater the internal friction angle (Li et al., 2018). During the *CU* test, the soil sample continued to dilate which results to the volume became larger, and the dry density became relatively smaller, which was the main reason for the change of the original cohesion and the internal friction angle.

**Fig 11:**
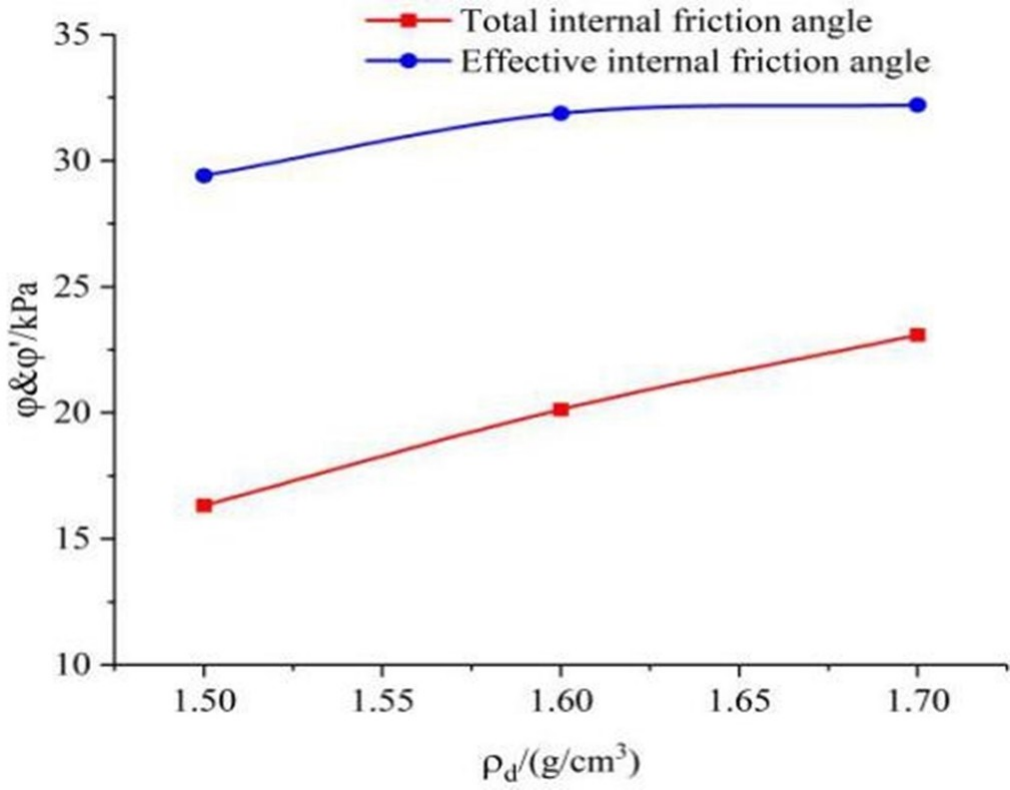
The curve of shear strength index *c* with dry density.

**Fig 12:**
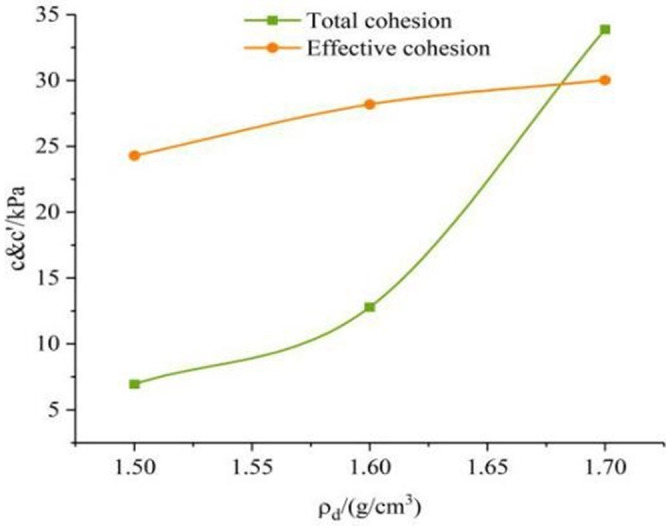
The curve of shear strength index *φ* with dry density.

The relationship between cohesion, internal friction angle of 0.04*mm/min* shear rate and dry density in Table 4 is shown in Figs 11 and 12. From the graph, it can be seen that cohesion and internal friction angle of saturated remolded loess samples increase with the increase of dry density. Because of the larger density of the sample, the pores between the particles are gradually reduced due to compaction, and the particles are more closely packed with each other, which causes the bite and the frictional force to increase accordingly between the particles, so the cohesive force increases significantly with the increase of dry density. As the dry density increases, the contact between the particles in the soil also tends to be tight, and the void ratio is relatively reduced. The moisture in the sample mostly remains in the manner of strongly binding the water film on the surface of the soil particles, and the strong absorbed water is relatively stable and cannot move, so the internal friction angle *φ* of the sample shows an increasing tendency as its dry density increases. The above shows that the internal friction angle does not increase infinitely with the increase of dry density, and there may be a critical value (Chen et al., 2005) [26], while the cohesion increases with the increase of dry density.

## 4 Conclusion

1. The deviatoric stress of specimens increases significantly at the same shear rate with an increase in dry density. The deviatoric stress-axial strain curves of the specimens at different dry densities are strain-hardening, and the hardening trend of the specimens increases with the increase of dry density. The stress-strain curves of the saturated remolded soil specimens show two forms of weakening and softening under a certain shear rate, and the softening is obvious under low confining pressure.
2. When dry density is equal to *ρ*_*d*_ = *1*.*5g/cm*^*3*^, an obvious critical strain rate, the shear strength of saturated remolded loess increases first, then decreases with an increase in shear rate and confining pressure. When dry density is equal to *ρ*_*d*_ = 1.6 *g/cm*^*3*^ and *ρ*_*d*_ = 1.7 *g/cm*^*3*^, the deviatoric stress increases first, then decreases andincreases with the increase of shear rate, which is different from that when dry density is equal to *ρ*_*d*_ = 1.5 *g/cm*^*3*^ when the shear rate is 0.4 *mm/min*.
3. When dry density is equal to 1.5 *g/cm*^*3*^, the cohesion decreases first and then increases with the increase of shear rate. When dry density is equal to *1*.*6 g/cm*^*3*^ and 1.7 *g/cm*^*3*^, the cohesive force first increases at 0.08 mm/min, then decreases and then increases with the increase of shear rate.
4. The cohesion and internal friction angle of remolded saturated loess samples increase with increasing dry density. When the dry density is small, the increasing trend of shear strength index *φ* is larger, but with the increase of dry density, the increasing trend slows down, while the cohesion raises with the increase of dry density as a whole.

## List of symbols

**Table:** 

A	pore pressure coefficients A
B	pore pressure coefficients B
*p*	the center coordinate of the circle of molar stress
*q*	the radius of the mohr’ s stress circle
*ρ*_*d*_	dry density
